# Atom Probe Tomographic Imaging of PbS Quantum Dot Formation on Neodymium Clusters in Silicate Glasses

**DOI:** 10.1038/s41598-019-46574-1

**Published:** 2019-07-11

**Authors:** Won Ji Park, Ju Eun Kim, Ho Jeong Lee, Chan Gyung Park, Jong Heo

**Affiliations:** Department of Materials Science and Engineering, Pohang University of Science and Technology (POSTECH), Pohang, Gyeongbuk 37937 Republic of Korea

**Keywords:** Characterization and analytical techniques, Imaging techniques

## Abstract

The first 3-D direct observation of clusters of Nd oxide inside silicate glasses was achieved using atom probe tomography. Three-dimensional elemental maps of major chemical elements in glasses such as Si, Al, Zn and O showed no evidence of regions that had concentrations higher than the average values, whereas the Nd aggregated into regions of high concentration. Elemental maps of Nd and Pb recorded from the glasses containing PbS QDs showed highly-concentrated areas of both elements at the same locations; this result indicates that PbS QDs formation started in association with the Nd clusters.

## Introduction

Rare-earth (RE)-doped glasses have been commercially used as signal amplifiers for optical communication and solid-state lasers that exploit the radiative f-f electronic transitions of trivalent RE ions^1–5^. One of the fundamental difficulties associated with emission by RE ions is that when RE ions assemble into clusters, concentration quenching occurs, in which neighboring ions exchange energy non-radiatively^6–9^. Many spectroscopic analyses have provided indirect evidence of RE clustering^10,11^ but direct observation has not been reported.

Quantum dots (QDs) can provide emissions from mid-infrared to visible wavelength^12–19^. The optical and electronic properties of QDs depend on their size, so many attempts to realize the precise control of QDs size^20–26^ have been reported. In particular, in glasses that include RE ions, QDs form near clusters of RE oxides that appeared to provide nucleation sites^27–29^. Distribution of elements analyzed from electron energy loss spectroscopy (EELS) suggested the presence of RE clusters. However, direct evidence of the presence of the RE-oxide clusters in conventional oxide glass is lacking, and their functions in nucleation of QDs in glasses are not known.

Atom probe tomography (APT) can provide single-atom sensitivity and fine spatial resolution (~0.1 nm in depth) of the distribution of chemical elements in three dimensions^30,31^. Local electrode atom probe (LEAP) uses an additional electrode to reduce the laser damage during analyses of insulating materials. Statistical tools including iso-surface image and maximum separation method can provide additional means to detect clusters or ordered phases with sizes smaller than a few nanometers^32,33^. We have employed APT to visualize the presence of RE clusters in silicate glasses. We also propose how RE clusters affect nucleation and growth of QDs in glasses.

## Results

### Specimen preparation using a dual beam focused ion beam method

**Figure 1:**
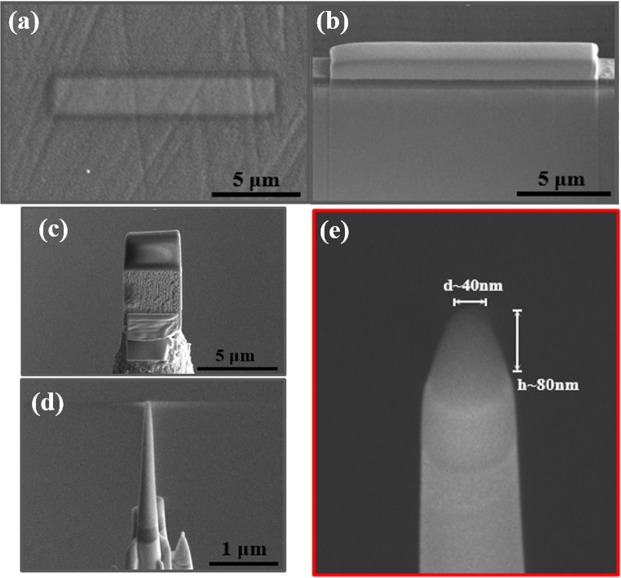
Procedures for preparation of tip-shaped glass specimen by using focused ion beam (FIB; FEI, Helios Nano-Lab 650) milling. (**a**) Pt deposition (~15 μm in length) to protect the surface of the specimen, (**b**) Cross-section view of a thin slab detached from the glass specimen, (**c**) Glass thin slab attached to tungsten tip, (**d**) Further milling into a needle shape, (**e**) Final specimen with 40-nm diameter and 80-nm length.

Specimens for APT analysis were prepared by a dual-beam focused ion beam (FIB; FEI, Helios Nano-Lab 650) method using the standard lift-out technique designed for insulating materials (Fig. 1)^34–36^. First, Pt coating was sputtered (CRESSINGTON SPUTTER COATER 208HR) on the surface of glass specimens to avoid surface damage by Ga^+^ ions (Fig. 1a). Then a thin slice of the specimen was lifted out of bulk glass material by several successive Ga^+^ ion-beam millings, including trench milling of H-beam type (Fig. 1b). This specimen with ~2-μm width was repositioned on the tungsten tip (Fig. 1c) and further milled into a needle shape (Fig. 1d) with diameter of ~40 nm and length of ~80 nm, that was finally loaded into the atom probe (Fig. 1e).

### Three-dimensional elemental mapping and computational analyses

**Figure 2:**
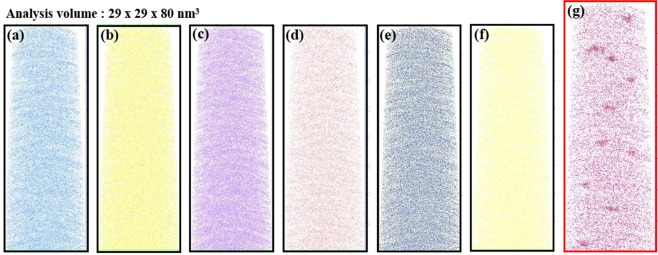
Three-dimensional elemental distribution maps for (**a**) Si, (**b**) O, (**c**) Zn, (**d**) Al, (**e**) S, (**f**) Pb and (**g**) Nd atoms. Volumes of glass used for analyses were ~29 × 29 × 80 nm. Every dots corresponds to each atom and the position of an atom represents the actual location in the glass matrix.

First, APT analysis was performed on as-prepared glass with the composition 50SiO_2_–5Al_2_O_3_–25NaO–10BaO–8ZnO–2ZnS–0.8PbO–5Nd_2_O_3_ (in mol%). In tomographic reconstructions (Fig. 2), all elements (Si, O, Zn, Al, S and Pb; Fig. 2a–f) except Nd (Fig. 2g) were distributed homogeneously. Nd concentration ([Nd]) was high in many regions. This is the first visual three-dimensional (3D) mapping of RE clusters in a glass. Clustering of RE ions in solid matrices has been considered as a major source of the energy transfers that degrade the emission efficiencies of REs. For example, fluorescence intensity of the Tb^3+^: ^5^D_3_ → ^7^F_5_ transition decreases as [Tb^3+^] in the glass increases^37^. Indirect evidence such as decrease in fluorescent lifetimes and fluorescent intensities of RE ions at certain energy levels have been used to propose the presence of clustering, but this is the first 3-D direct observation of regions with RE ion concentration higher than those expected from the homogeneous distribution.

**Figure 3:**
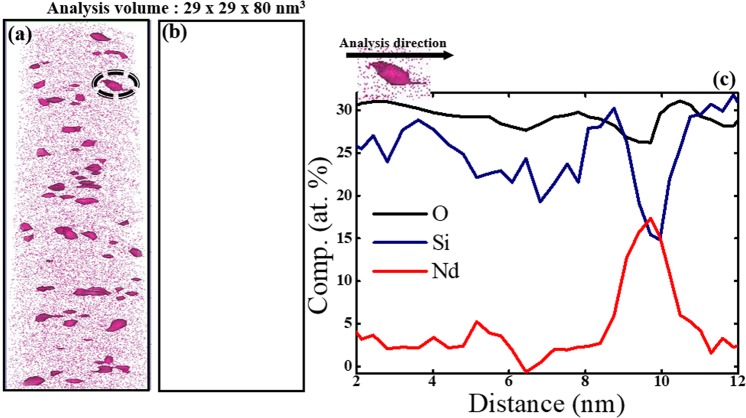
Isolated atom distribution image of (**a**) Nd and (**b**) Si obtained from the iso-surface analysis with 1.40 at.% each atom. (**c**) Concentration profile of each element (Si, O, Nd) in a cluster of ~3-nm diameter.

We further performed computational analysis using the iso-surface imaging method to visualize areas of high [Nd] clearly. The 1.40% Nd-iso surface analysis shows clear images of Nd-rich regions inside the matrix (Fig. 3a). A similar analysis for Si did not reveal any images of clustered areas, because Si was distributed homogeneously in the specimen so the analysis did not form a closed surface (Fig. 3b). The elemental concentration profile (Fig. 3c) recorded from one Nd cluster indicated that [Nd] atoms increased to ~18 at.% across ~3 nm in diameter. [Si] in the cluster decreased, but [O] remained constant at ~30 at.%.

**Figure 4:**
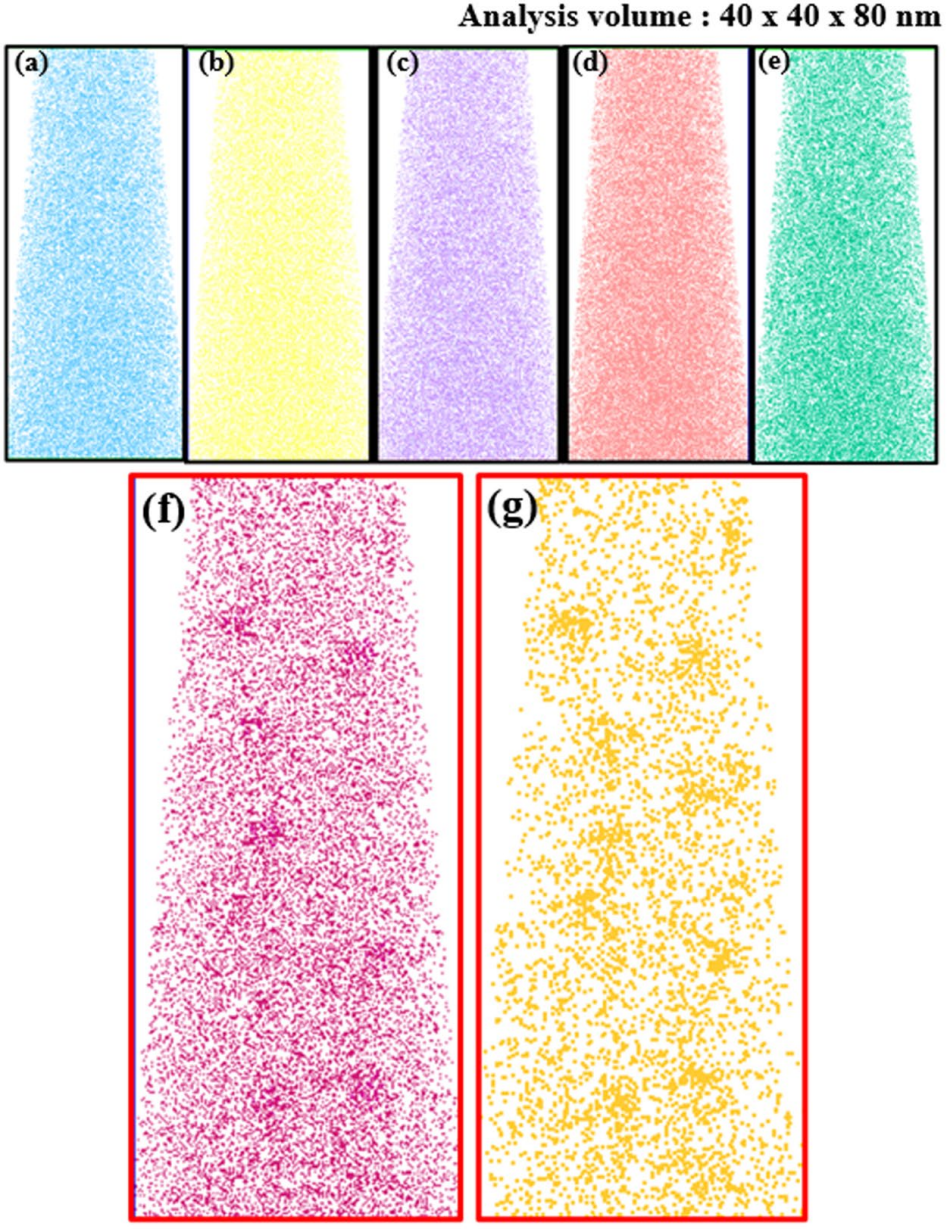
Three-dimensional elemental distribution maps for (**a**) Si, (**b**) O, (**c**) Zn, (**d**) Al **(e**) S, (**f**) Nd and (**g**) Pb atoms in glasses after heat treatment at 500 °C for 30 h. Volumes of glass used for analyses were ~40 × 40 × 80 nm.

Afterwards, glass was heat-treated at 500 °C for 30 h to precipitate PbS QDs. 3D distributions (Fig. 4a) of each element were obtained from a volume of approximately 40 × 40 × 80 nm in silicate glass containing 5.0 mol.% Nd_2_O_3_. Most of the elements (e.g., Si, O, Zn, Al, S atoms) that form the glass matrix were uniformly distributed (Fig. 4a–e). Some clustered areas had high [Nd] (Fig. 4f) and high [Pb] (Fig. 4g), but some Nd and Pb remained in the glass matrix. Regions of high [Nd] concentration (Fig. 4f) appear to overlap regions of high [Pb] (Fig. 4g).

**Figure 5:**
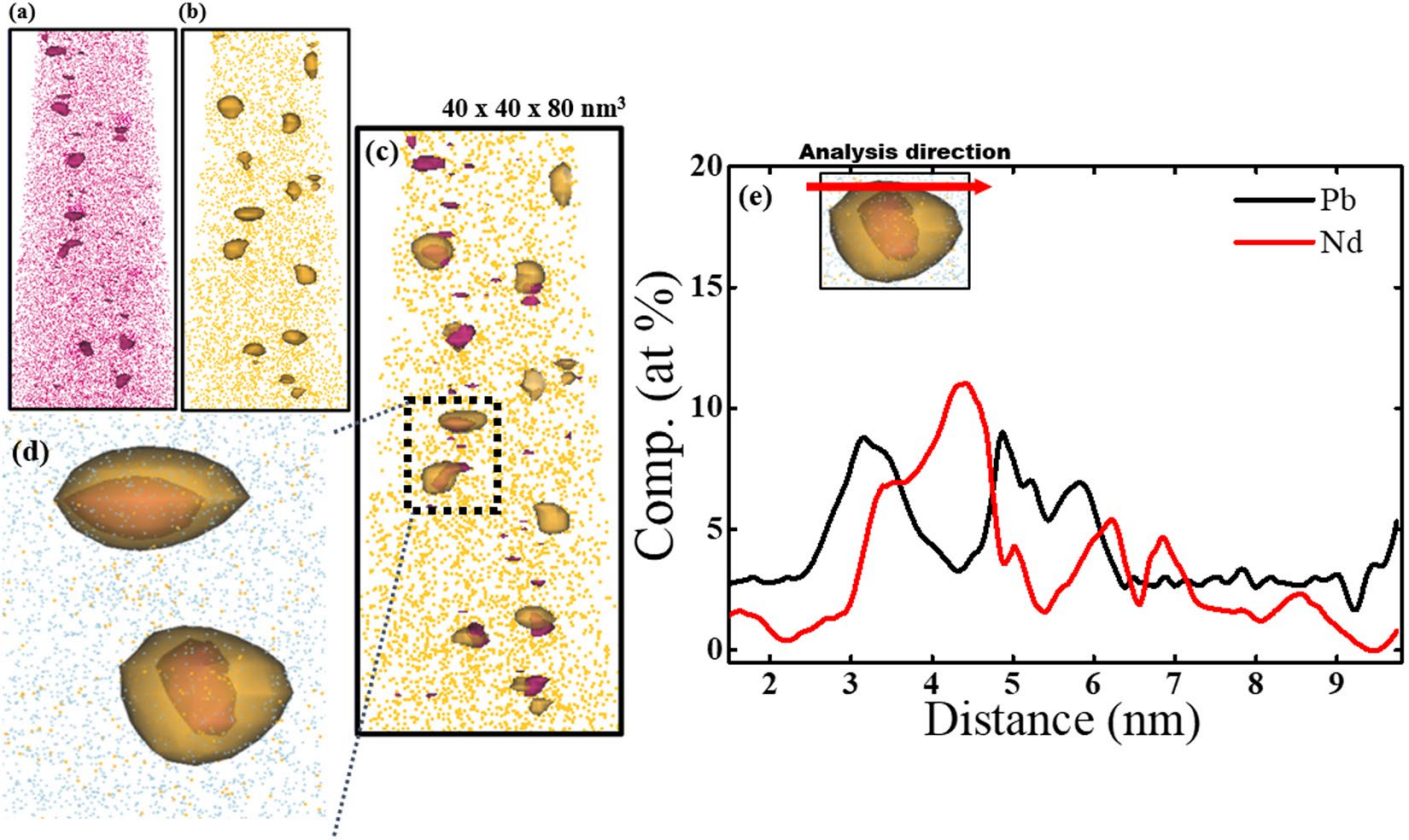
Isolated atom distribution image obtained from iso-surface concentration analyses of (**a**) Nd with 1.40 at.% Nd, (**b**) Pb with 1.40 at.% Pb and (**c**) graphical combination of (**a**,**b**). (**d**) Magnified image of two PbS QDs in (**c**,**e**) concentration profiles of Pb and Nd in a QDs with ~4-nm diameter.

We also conducted iso-surface compositional analyses for areas of clustered Nd and Pb. Results of the analyses with 1.40% Nd iso-surface concentration contour showed Nd-rich regions (Fig. 5a) similar to those in Fig. 3a. In addition, areas with high [Pb] (i.e., PbS QDs) were detected in glasses (Fig. 5b). In the same analysis, Si and Al atoms did not show clustering. Combination of Fig. 5a,b proved that Nd clusters almost coincide with the location of PbS QDs (Fig. 5c); this result suggests that the growth of PbS QDs is closely related to the presence of Nd clusters (Fig. 5d) and it is consistent with the results of electron energy loss spectroscopy (EELS) (Fig. S1)^28^. The elemental concentration profile along one QD showed that [Pb] increased to ~10 at.%, and [Nd] increased to 12 at.% (Fig. 5e).

### Nd cluster analysis using the maximum separation method

We visualized Nd clusters in 3D elemental mapping (Fig. 2) and iso-surface images (Fig. 3). We also used the maximum-separation method. The value of χ^2^ is usually determined from the distribution of Nd inside the matrix: χ^2^ = 0 suggests a random distribution; χ^2^ > 0 indicate that clusters exist^38–40^. The glass specimen that contained 5.0 mol.% of Nd_2_O_3_ had χ^2^ ~0.93 before heat treatment. This result is another evidence of a non-uniform and heterogeneous distribution of Nd in the glass matrix.

**Figure 6:**
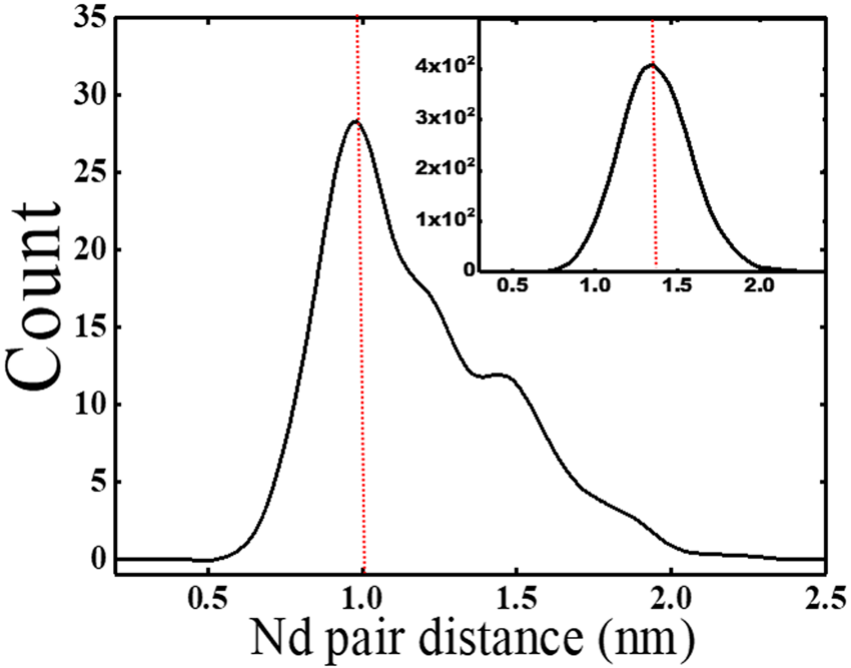
First nearest neighbor (1NN) distance D_max_ distribution of Nd inside clusters to obtain the value of D_max_ for the maximum separation method. Inset: 1NN distance of Nd in glass matrix.

**Figure 7:**
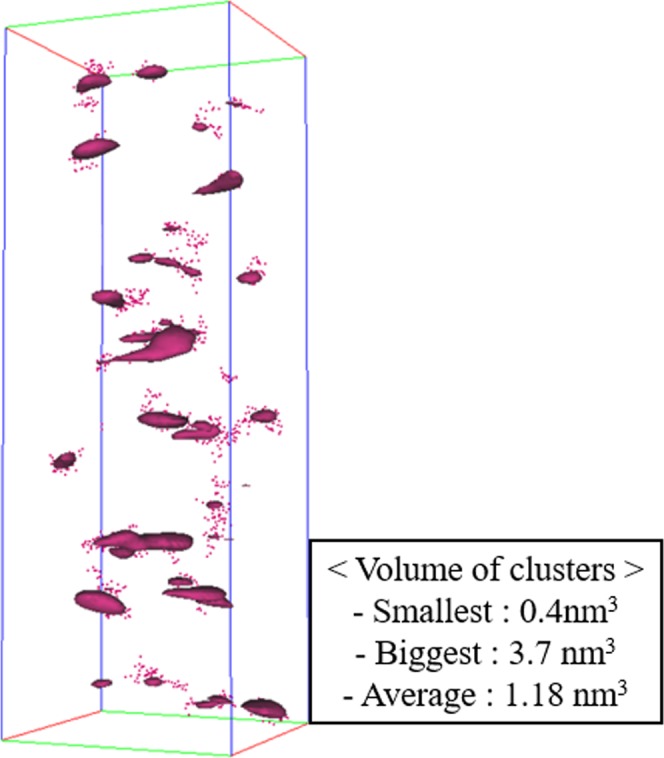
Analyzed Nd clusters by maximum separation algorithm with appropriate parameters. Inset table: compositional difference between clusters and glass matrix.

Afterwards, the Nd-Nd nearest-neighbor analysis between cluster and matrix was obtained from Fig. 6 to determine the nearest neighbor distance *D*_max_ between Nd atoms. Evaluation considered 14471 blocks of 100 atoms. *D*_max_ was ~1.0 nm in the clusters ~1.5 nm in the glass matrix. Other parameters considered are *Order*, which is the number of nearest Nd atoms around a specific Nd atom; *N*_min_, which is the minimum size of cluster that can be considered as a cluster; and *E*, which is the erosion distance at which an atom can be considered to have moved from the cluster to the matrix^41^. Numbers used for the cluster analysis were *D*_max_ = 1.0, *Order* = 6, *N*_min_ = 10 and *E* = 1.0 nm; analysis detected 81 Nd clusters in Fig. 7a, with volumes of 0.40–3.7 nm^3^ (average 1.18 nm^3^). Average [Nd] within the clusters was 56.3 at.%, which is ~19 times higher than the average [Nd] = 3.3 at.% in the glass matrix.

Nd^3+^ ions are surrounded by an average of seven oxygens both before and after the formation of PbS QDs in glasses^29^. Therefore, clusters of high [Nd] (Figs 2 and 4) are most probably made of NdO_x_ polyhedra rather than of Nd-Nd bonds. Precipitation of PbS QDs occurs in association with Nd-O clusters.

We have demonstrated the presence of Nd clusters in silicate glasses by using atom probe tomography and 3D elemental mapping. Distributions of the major chemical elements that constitute the (e.g., Si, Al, O) showed no evidence clustering. When the glasses were heat-treated at 500 °C for 30 h to precipitate the PbS QDs, areas of high [Nd] and [Pb] formed in the glasses, and closely coincide with each other; this result indicates that formation of PbS QDs most probably started at the Nd clusters.

## Method

### Glass preparation

A base glass with a nominal composition of 50SiO_2_–5Al_2_O_3_–25NaO–10BaO–8ZnO–2ZnS–0.8PbO (in mol%) and a Nd-glass with an additional 5 mol% of Nd_2_O_3_ were prepared using a conventional melt-quenching process. Starting powders were mixed and melted in an alumina crucible at 1340 °C for 30 min. Each melt was poured onto a brass mold that had been preheated at 380 °C, then was quenched by pressing with another brass plate. Resulting glasses were annealed at 380 °C for 2 h to eliminate residual thermal stress. Specimens were polished to ~150-μm thickness, then used as a source for fabrication by FIB of specimens for APT.

### Atom Probe Tomography (APT)

APT analysis (CAMECA, LEAP4000X HR) was performed using a pulsed UV laser (λ = 355 nm) and each atom was detached from the surface by irradiating the laser beam with 100-pJ pulse energy at a repetition rate of 200 kHz. An additional local electrode with a range of 2–15 kV was used to avoid the specimen damage by the laser beam. IVAS software (3.6.10a version) by CAMECA Instruments was used to reconstruct and visualize APT results. Cluster formation was identified from the iso-surface concentration. The maximum-separation algorithm was used to distinguish the clusters from the matrix, and the corresponding differences between the matrix and cluster compositions were calculated.

## Supplementary information

Atom Probe Tomographic Imaging of PbS Quantum Dot Formation on Neodymium Clusters in Silicate Glasses
