# Supplementary material for: Atom Probe Tomographic Imaging of PbS Quantum Dot Formation on Neodymium Clusters in Silicate Glasses
Source: Sci Rep. 2019 Jul 11;9:10029. doi: 10.1038/s41598-019-46574-1 (PMC6624314; doi:10.1038/s41598-019-46574-1)
Supplement: Supplementary file 1 — Atom Probe Tomographic Imaging of PbS Quantum Dot Formation on Neodymium Clusters in Silicate Glasses [file 41598_2019_46574_MOESM1_ESM.docx]

**Atom Probe Tomographic Imaging of PbS Quantum Dots Formation on Neodymium Clusters in Silicate Glasses**

Won Ji Park^+^, Ju Eun Kim, Ho Jeong Lee, Chan Gyung Park, Jong Heo^*^

*Department of Materials Science and Engineering, Pohang University of Science and Technology (POSTECH), Pohang, Gyeongbuk 37937, Republic of Korea*

*Correspondence and requests for materials should be addressed to J. Heo

(email: jheo@postech.ac.kr)


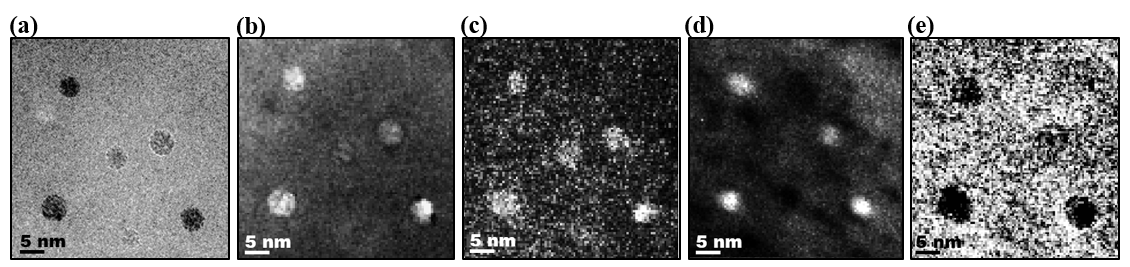


**Figure S1.** Electron energy loss spectroscopy (EELS) results of silicate glasses with 5 mol% Nd_2_O_3_ after heat treatment at 500^o^C for 30hrs; (a) the micrograph of PbS QDs and the elemental distribution of (b) Pb, (c) S, (d) Nd and (e) Si.

Figure S1 shows results of the electron energy loss spectroscopy (EELS) for elements in silicate glasses containing 5.0 mol% Nd_2_O_3_. The areas with relatively high (low) concentrations appeared brighter (darker) than remainder. The QDs are rich in Pb, S and Nd, but depleted in Si. Therefore, QDs made inside silicate glasses are PbS and Nd ions exist preferentially inside these QDs.
